# Risk factors for self-reported arm lymphedema among female breast cancer survivors: a prospective cohort study

**DOI:** 10.1186/s13058-014-0414-x

**Published:** 2014-08-22

**Authors:** Kayo Togawa, Huiyan Ma, Jane Sullivan-Halley, Marian L Neuhouser, Ikuyo Imayama, Kathy B Baumgartner, Ashley Wilder Smith, Catherine M Alfano, Anne McTiernan, Rachel Ballard-Barbash, Leslie Bernstein

**Affiliations:** 1 Department of Preventive Medicine, Keck School of Medicine, University of Southern California, 1975 Zonal Avenue, Los Angeles, 90032 CA USA; 2 Division of Cancer Etiology, Department of Population Sciences, Beckman Research Institute, City of Hope, 1500 E Duarte Road, Duarte, 91010 CA USA; 3 Division of Public Health Sciences, Fred Hutchinson Cancer Research Center, 1100 Fairview Avenue North, Seattle, 98109 WA USA; 4 Department of Epidemiology & Population Health, University of Louisville, 485 East Gray Street, Louisville, 40202 KY USA; 5 Division of Cancer Control and Population Sciences, National Cancer Institute, 9609 Medical Center Drive, Bethesda, 20892 MD USA

## Abstract

**Introduction:**

Lymphedema is a potentially debilitating condition that occurs among breast cancer survivors. This study examines the incidence of self-reported lymphedema, timing of lymphedema onset, and associations between sociodemographic, clinical and lifestyle factors and lymphedema risk across racial-ethnic groups using data from a multicenter, multiethnic prospective cohort study of breast cancer survivors, the Health, Eating, Activity and Lifestyle Study.

**Methods:**

A total of 666 women diagnosed with breast cancer staged as *in situ*, localized or regional disease at ages 35 to 64 years were recruited through the Surveillance, Epidemiology, and End Results registries in New Mexico (non-Hispanic white and Hispanic white), Los Angeles County (black), and Western Washington (non-Hispanic white) and followed for a median of 10.2 years. We evaluated sociodemographic factors, breast cancer- and treatment-related factors, comorbidities, body mass index (BMI), hormonal factors, and lifestyle factors in relation to self-reported lymphedema by fitting Cox proportional hazards models, estimating hazard ratios (HR) and 95% confidence intervals (CI).

**Results:**

Over the follow-up period, 190 women (29%) reported lymphedema. The median time from breast cancer diagnosis to onset of lymphedema was 10.5 months (range: 0.5 to 134.9 months). Factors independently associated with lymphedema were total/modified radical mastectomy (versus partial/less than total mastectomy; HR = 1.37, 95% CI: 1.01 to 1.85), chemotherapy (versus no chemotherapy; HR = 1.48, 95% CI: 1.09 to 2.02), no lymph nodes removed (versus ≥10 lymph nodes removed; HR = 0.17, 95% CI: 0.08 to 0.33), pre-diagnostic BMI ≥30 kg/m^2^ (versus BMI <25 kg/m^2^; HR = 1.59, 95% CI: 1.09 to 2.31), and hypertension (versus no hypertension; HR = 1.49, 95% CI: 1.06 to 2.10). After adjusting for demographics and breast cancer- and treatment-related factors, no significant difference in lymphedema risk was observed across racial/ethnic groups. Analyses stratified by race/ethnicity showed that hypertension and chemotherapy were lymphedema risk factors only for black women.

**Conclusions:**

Breast cancer patients who have undergone extensive surgery or extensive lymph node dissection, or who have a higher BMI should be closely monitored for detection and treatment of lymphedema. Further studies are needed to understand the roles of chemotherapy and hypertension in the development of lymphedema.

**Electronic supplementary material:**

The online version of this article (doi:10.1186/s13058-014-0414-x) contains supplementary material, which is available to authorized users.

## Introduction

Lymphedema is a relatively common and potentially debilitating condition in which there is an excessive accumulation of lymphatic fluid in the arm or hand. It develops in approximately 20% of women after treatment for breast cancer [1],[2]; it can occur as a result of damage to the lymphatic system from breast cancer treatment such as axillary lymph node dissection or axillary radiotherapy [3]. The use of sentinel lymph node biopsy, which avoids unnecessary axillary lymph node surgery in patients with pathologically negative nodes, has reduced the risk of lymphedema [4]; however, the risk of lymphedema has not been completely eliminated [5]. About 25% of patients who undergo sentinel lymph node biopsy have positive nodes, for which patients undergo axillary treatment [6]. Thus, many breast cancer patients remain at risk of lymphedema.

Although lymphedema is not considered life threatening, its consequences include cosmetic deformity, physical discomfort, and upper extremity disability [7]. Lymphedema also increases the risk of cellulitis, lymphangitis, and occasionally lymphangiosarcoma [8]-[10]. No known definitive cure for lymphedema is available and thus, affected women live with lymphedema for many years. Given the negative impact on quality of life [11]-[14] and the potentially higher medical cost of managing lymphedema and treating lymphedema-induced conditions [8], preventive measures are desired.

Many studies have evaluated a variety of demographic, health, and clinical characteristics in relation to lymphedema [12],[15]-[32]; however, the results vary widely, possibly due to differences in study design, statistical power, analytic methods, measures used to define lymphedema, demographic characteristics, or length of follow-up. Furthermore, the majority of prior studies lacked racial/ethnic diversity [18],[19],[22],[28],[29] or long-term follow-up data [16]-[19],[24],[27],[28],[30]-[32]; data from long-term prospective cohort studies in a diverse population remain scarce. The Health, Eating, Activity, and Lifestyle (HEAL) Study, has followed a cohort of breast cancer survivors for more than 10 years and consists of non-Hispanic white women, Hispanic women, and black women. Here, we assess the incidence of self-reported lymphedema, timing of lymphedema onset, and associations with breast cancer-related and treatment-related factors, sociodemographic factors, comorbidities, hormone-related factors, and lifestyle factors across three racial/ethnic groups.

## Materials and methods

### Study setting, subjects, and recruitment

**Figure 1:**
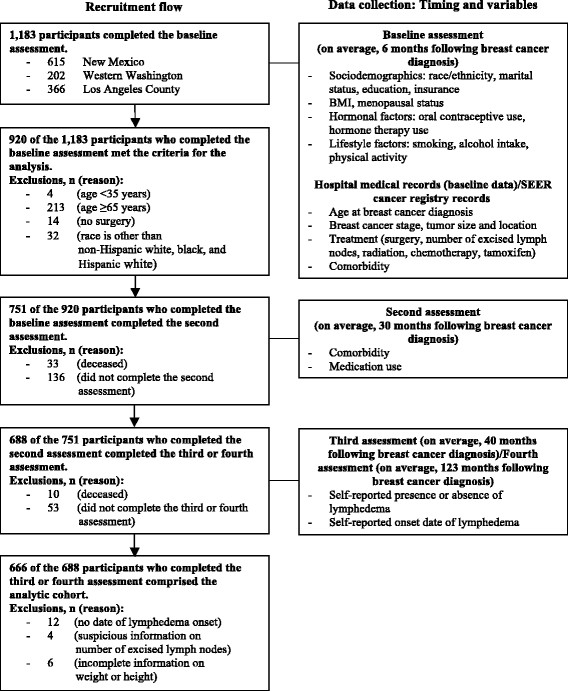
**Recruitment flow and data collection.**

The aims, study design, and recruitment procedures of the HEAL Study have been published previously [33],[34]. Briefly, the HEAL Study is a multicenter, multiethnic prospective breast cancer cohort study. Women diagnosed with first primary *in situ* or Stage I to IIIA invasive breast cancer between 1995 and 1999 were recruited into the HEAL Study through the Surveillance, Epidemiology, and End Results (SEER) registries in three regions of the United States: New Mexico, Western Washington, and Los Angeles County, California. The age ranges studied varied by study site with women aged less than 92 years recruited in New Mexico, women aged 40 to 65 years recruited in Western Washington, and women aged 35 to 64 years recruited in Los Angeles County. A total of 1,183 women completed up to five assessments over 10 years of follow-up. Four of those assessments were used for this study. The first assessment (baseline assessment) was administered in person within the first year (on average, 6 months) after a woman’s diagnosis (Figure 1). The second assessment was administered, on average, 30 months after a woman’s diagnosis (30-month assessment). The 30-month assessment was administered via in-person interview or self-completed questionnaire. The third assessment was administered, on average, 40 months after a woman’s diagnosis (40-month assessment) by telephone interview or mailed questionnaire in New Mexico, by mailed questionnaire plus telephone follow-up in Western Washington, and by telephone interview in Los Angeles County. The last assessment was administered, on average, 123 months after a woman’s diagnosis (123-month assessment) by telephone interview in New Mexico and Los Angeles County and by mailed questionnaire or telephone interview in Western Washington.

In the present study, we excluded 217 women who were younger than 35 years (n = 4) or older than 64 years (n = 213) in order to provide similar age distributions across study sites. We also excluded 14 women who did not receive any type of surgery; and 32 women whose racial/ethnic classification was other than Hispanic white, non-Hispanic white, or black, leaving 920 women. A total of 751 (82%) of the 920 women completed the 30-month assessment. A total of 688 (92%) of the 751 women completed either the 40-month assessment or the 123-month assessment (496 women completed both the 40-month and the 123-month assessments, 163 women completed only the 40-month assessment, 29 women completed only the 123-month assessment). We then excluded 22 women with incomplete or questionable data on onset date of lymphedema (n = 12), number of excised lymph nodes (n = 4), or body mass index (BMI) (n = 6). The final analytic cohort consisted of 666 women; 666 (72%) of the eligible cohort had complete data for this analysis (Figure 1).

We obtained informed consent from all participants at each assessment. The study was approved by the institutional review boards at the University of Southern California, University of New Mexico, Fred Hutchinson Cancer Research Center, University of Louisville, and Beckman Research Institute of City of Hope in accord with assurances filed with and approved by the U.S. Department of Health and Human Services.

### Data collection

#### Arm lymphedema

Participants provided information on lymphedema at the 40-month and 123-month assessments. To determine the presence of lymphedema, we first presented our definition of lymphedema to study participants: ‘Sometimes the arm on the side on which you had breast cancer becomes swollen because of an accumulation of fluid in your arm. This is called lymphedema. Please do not confuse this with the temporary swelling that occurs after surgery’. We then asked the following ‘yes’ or ‘no’ question: ‘Have you experienced lymphedema in your arm at any time since your breast cancer diagnosis?’ For women who answered ‘yes’, we also asked when they first experienced lymphedema symptoms (month and year), and whether they were still experiencing lymphedema at the time of assessment. Lymphedema occurring within one year of breast cancer diagnosis was considered early-onset lymphedema, whereas that occurring more than one year after diagnosis was considered late-onset lymphedema.

#### Breast cancer-related and treatment-related factors

We obtained clinical data on diagnosis date, age at diagnosis, disease stage (SEER staging), treatment types (surgeries, radiation therapy, chemotherapy), tumor size, cancer location, and number of excised lymph nodes from SEER cancer registry records and by abstracting participants’ hospital medical records. Tamoxifen use at or prior to baseline was assessed using both hospital medical records and self-report.

#### Sociodemographic and lifestyle factors

The baseline assessment captured sociodemographic information such as race/ethnicity, marital status, educational status, and insurance status. The baseline assessment also captured information on history of smoking, alcohol intake in the year prior to diagnosis, and sports and recreational physical activity in the year prior to diagnosis. We calculated pack-years of smoking as the number of packs of cigarettes per day times the number of years the woman smoked, grams of alcohol consumed per day, and metabolic equivalent task (MET) hours of sports and recreational physical activity based on the Compendium of Physical Activities compiled by Ainsworth *et al.*[35].

#### Health-related and hormone-related factors

Information on comorbid medical conditions such as diabetes, hypertension, and arthritis, was collected through self-report and hospital records. The number of women having each condition and the proportion of women who were identified in both sources, by self-report only and by hospital records only, were as follows: hypertension - 198 women, 63%, 28%, 9% respectively; diabetes - 59 women, 53%, 32%, 15%, respectively; and arthritis - 186 women, 18%, 78%, 3%, respectively. Charlson Comorbidity Index [36] was calculated based on data from hospital medical records [37]. We did not count any carcinoma as part of the Charlson Comorbidity Index. Participants also reported information on height at age 18 years and weight five years before diagnosis (black women in Los Angeles County) or weight one year before diagnosis (Hispanic women in New Mexico and non-Hispanic white women in New Mexico and Western Washington) at the baseline assessment. BMI prior to diagnosis was calculated as weight (kg) divided by the square of height (m^2^). The baseline assessment also captured information on oral contraceptive use and postmenopausal hormone replacement therapy use before breast cancer diagnosis, and menopausal status at diagnosis. Menopausal status was determined based on the following questionnaire data: age, date of last menstruation, and hysterectomy and oophorectomy status [38].

### Statistical analysis

**Figure 2:**
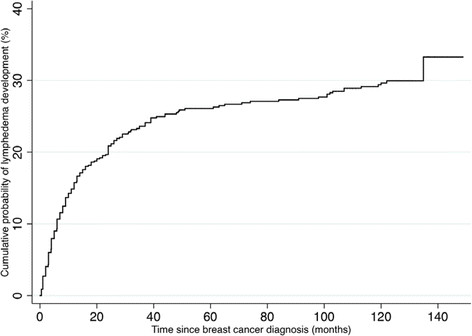
**Kaplan-Meier cumulative incidence estimate of self-reported arm lymphedema.**

Descriptive statistics by race/ethnicity were obtained for breast cancer-related and treatment-related factors, sociodemographic factors, health-related factors, hormonal factors, and lifestyle factors (Table S1 in Additional file 1). Time to onset of lymphedema was calculated as the time from breast cancer diagnosis until self-reported first lymphedema occurrence; we used these data to generate a cumulative incidence curve (Figure 2).

To identify factors associated with self-reported lymphedema, we fit Cox proportional hazards models and obtained estimates of the hazard ratio (HR) and its 95% confidence interval (CI) using time since diagnosis as the time scale [39]. Women were followed from date of breast cancer diagnosis to date of first lymphedema occurrence or until date of last follow-up. One hundred fifty-six women who died or were lost to follow-up between the 40-month and the 123-month assessments contributed follow-up only through the 40-month assessment because their lymphedema status after the 40-month assessment was unknown. We considered the following variables as potential covariates in our multivariate models: breast cancer disease stage (*in situ*, localized, regional), tumor size (smaller than 10, 10 to 19, 20 mm or greater, missing), tumor location (nipple/central portion or upper/lower inner quadrant, upper/lower outer quadrant, overlapping lesion, axillary tail or not specified), surgery type (partial or less than total mastectomy or unspecified surgery, total or modified radical mastectomy), reconstructive surgery (yes, no), number of excised lymph nodes (0, 1 to 9, 10 or more), radiation therapy (yes, no), chemotherapy (yes, no), tamoxifen (yes, no), marital status (married, widowed, divorced or separated, never married), education status (high school or less, some college, college degree, graduate studies), medical insurance (yes, no), BMI prior to diagnosis (less than 25 (underweight or normal), 25 to 29.9 (overweight), and 30 kg/m^2^ or above (obese)), menopausal status at diagnosis (premenopausal, postmenopausal, unknown), hypertension at or prior to diagnosis (yes, no), diabetes at or prior to diagnosis (yes, no), arthritis at or prior to diagnosis (yes, no), and Charlson Comorbidity Index (0, 1 to 2), oral contraceptive use prior to diagnosis (yes, no), estrogen use prior to diagnosis (yes, no, missing), progestin use prior to diagnosis (yes, no, missing), pack-years of smoking (less than 0.05, 0.05 to 5.3, 5.4 to 20.5, more than 20.5), alcohol intake in the year prior to diagnosis (less than 1, 1 to 6, more than 6 grams per day), physical activity during the year prior to diagnosis (less than 0.5, 0.5 to 20, more than 20 MET hours per week). Age at diagnosis (35 to 44, 45 to 49, 50 to 54, 55 to 59, 60 to 64 years) and race/ethnicity (non-Hispanic white (Western Washington, New Mexico), black (Los Angeles County), Hispanic white (New Mexico)) were considered design variables and were included in all models.

Each variable was added to an age- and race/ethnicity-adjusted model and a likelihood ratio test was performed to test whether the variable significantly improved the model fit. Postmenopausal estrogen use and progestin use were examined among postmenopausal women only. In analyses of postmenopausal women and menopausal status, age was treated as continuous. All variables with a *P* value less than 0.05 based on the likelihood ratio test were then added to an age- and race/ethnicity-adjusted model one at a time in order from the variable with the smallest *P* value to the variable with largest *P* value to examine whether the addition of each variable improved the fit of the model. Only the variables that significantly improved the model were kept in the final model. The final multivariable model included race/ethnicity, age at diagnosis, surgery type, number of excised lymph nodes, chemotherapy, BMI and hypertension. We also tested for exposure-associated trends in risk of lymphedema for variables represented by ordinal values by fitting the original values in our model and testing whether the coefficient associated with that covariate differed from zero. In addition, we tested interactions between variables included in the final multivariable model by creating an interaction term and using a likelihood ratio test. We considered a two-sided *P* value less than 0.05 as statistically significant.

In exploratory analyses, we evaluated whether the association with lymphedema for each of the variables in the final multivariable model differed by timing of lymphedema onset; to do this, we created an interaction term using a time-dependent indicator for follow-up (12 months or less, longer than 12 months). We then performed a likelihood ratio test to compare the interaction model to the model without the interaction term. We also assessed the association of each risk factor with the development of lymphedema within each racial/ethnic group.

Finally, we performed sensitivity analyses by excluding women with *in situ* breast cancer and reevaluating the final multivariable model; we also reran analyses censoring women who developed a recurrence or new primary breast cancer at the time of these events.

All analyses were performed using the STATA software (Version 12; StataCorp LP, College Station, TX, USA).

## Results

### Characteristics of study population

The study population consisted of three racial/ethnic groups: 371 non-Hispanic white women (n = 225 (New Mexico), n = 146 (Western Washington)), 226 black women (Los Angeles County), and 69 Hispanic women (New Mexico). Participant characteristics by race/ethnicity are shown in Table S1 in Additional file 1. The mean age at breast cancer diagnosis was 51.5 years (standard deviation = 7.2). Approximately 23% of the participants had *in situ* breast cancer, 52% had localized disease, and 25% had regional disease. Prior to breast cancer diagnosis, approximately 33% of the women were overweight (BMI = 25.0 to 29.9 kg/m^2^) and 21% were obese (BMI ≥30 kg/m^2^).

The median length of follow-up since breast cancer diagnosis was 10.2 years (range: 2.4 to 12.4 years). During follow-up, 190 women (29%) reported lymphedema. The median time from breast cancer diagnosis to onset of lymphedema was 10.5 months (range: 0.5 to 134.9 months). The cumulative incidence of lymphedema was 15.8%, 20.9%, 26.1%, and 29.7%, at 1, 2, 5, and 10 year(s), respectively (Figure 2). One hundred nine (77%) of 141 women who reported lymphedema at the 40-month assessment completed the 123-month assessment. Among these 109 women, 63 (58%) women indicated that their lymphedema was still present at the time of the 123-month assessment.

### Age- and race/ethnicity-adjusted analyses

We present a series of hazard ratios for individual factors considered as potential risk factors for lymphedema in Table 1. When age at diagnosis and race/ethnicity were mutually adjusted, the oldest age group (60 to 64 years) had a lower risk of lymphedema than the youngest age group (35 to 44 years) (HR = 0.59, 95% CI: 0.35 to 0.97) and black women had a higher risk of lymphedema than non-Hispanic white women (HR = 1.62, 95% CI: 1.20 to 2.20). Women with *in situ* breast cancer had a substantially lower risk of lymphedema than women with localized breast cancer after adjusting for age at diagnosis and race/ethnicity (HR = 0.26, 95% CI: 0.15 to 0.45). Women with regional breast cancer were more likely to develop lymphedema than women with localized breast cancer; however the 95% CI included 1.0 (HR = 1.35, 95% CI: 0.99 to 1.85). We excluded disease stage from the final model since disease stage was strongly associated with treatment-related factors. In addition to disease stage, the following variables added significantly to the age- and race/ethnicity-adjusted model based on likelihood ratio test (*P* <0.05): tumor size, surgery type, number of excised lymph nodes, chemotherapy, tamoxifen, BMI, and hypertension. No statistically significant association was observed for other factors.

**Table 1:** **Participant characteristics and their associations with lymphedema among 666 female breast cancer survivors**

Characteristics	Categories	N at risk	LE (%)	HR (95% CI)^a^	HR (95% CI)^b^
Breast cancer and treatment					
Age at diagnosis (years)	35-44	123	43 (35.0)	1.00	1.00
	45-49	128	43 (33.6)	0.98 (0.64 - 1.50)	0.98 (0.63 - 1.52)
	50-54	175	46 (26.3)	0.80 (0.52 - 1.21)	0.79 (0.51 - 1.23)
	55-59	128	35 (27.3)	0.83 (0.53 - 1.30)	0.78 (0.49 - 1.23)
	60-64	112	23 (20.5)	0.59 (0.35 - 0.97)	0.61 (0.35 - 1.06)
	Per unit of age			0.98 (0.96 - 1.00)	0.98 (0.96 - 1.00)
				*P trend = 0.03*	*P trend = 0.04*
Disease stage	*In situ*	155	15 (9.7)	0.26 (0.15 - 0.45)	
	Localized	346	105 (30.4)	1.00	
	Regional	165	70 (42.4)	1.35 (0.99 - 1.85)	
Tumor size (mm)	<10	178	36 (20.2)	1.00	
	10-19	216	66 (30.6)	1.54 (1.02 - 2.32)	
	20+	208	76 (36.5)	1.85 (1.23 - 2.77)	
	Missing	64	12 (18.8)	0.80 (0.41 - 1.54)	
	Per mm			1.01 (1.00 - 1.02)	
				*P trend = 0.02*	
Cancer location	Nipple, central portion, upper/lower inner quadrant	143	44 (30.8)	1.15 (0.80 - 1.66)	
	Upper/lower outer quadrant	292	83 (28.4)	1.00	
	Overlapping lesion	147	44 (29.9)	1.07 (0.74 - 1.55)	
	Axillary tail, NOS	84	19 (22.6)	0.75 (0.46 - 1.24)	
Surgery type	Partial/less than total mastectomy/surgery, NOS	442	108 (24.4)	1.00	1.00
	Total mastectomy/modified radical mastectomy	224	82 (36.6)	1.52 (1.13 - 2.04)	1.37 (1.01 - 1.85)
Reconstructive surgery	No	533	150 (28.1)	1.00	
	Yes	105	33 (31.4)	0.98 (0.67 - 1.45)	
	Missing	28	7 (25.0)	-	
Number of excised lymph nodes	0	143	9 (6.3)	0.13 (0.06 - 0.25)	0.17 (0.08 - 0.33)
	1-9^c^	162	32 (19.8)	0.44 (0.30 - 0.64)	0.46 (0.31 - 0.68)
	10+	361	149 (41.3)	1.00	1.00
	Per lymph node			1.06 (1.04 - 1.08)	1.05 (1.03 - 1.07)
				*P trend <0.001*	*P trend < 0.001*
Radiation	No	270	73 (27.0)	1.00	
	Yes	396	117 (29.6)	1.21 (0.90 - 1.63)	
Chemotherapy	No	413	83 (20.1)	1.00	1.00
	Yes	253	107 (42.3)	2.28 (1.69 - 3.09)	1.48 (1.09 - 2.02)
Tamoxifen	No	382	96 (25.1)	1.00	
	Yes	284	94 (33.1)	1.58 (1.18 - 2.12)	
**Sociodemographic factors**					
Race/ethnicity	Non-Hispanic white (NM, WW)	371	88 (23.7)	1.00	1.00
	Black (LA)	226	82 (36.3)	1.62 (1.20 - 2.20)	1.16 (0.83 - 1.61)
	Hispanic white (NM)	69	20 (29.0)	1.19 (0.73 - 1.94)	1.04 (0.63 - 1.70)
Marital status	Married	445	123 (27.6)	1.00	
	Widowed	34	10 (29.4)	1.25 (0.64 - 2.43)	
	Divorced/separated	140	46 (32.9)	1.20 (0.86 - 1.69)	
	Never married	47	11 (23.4)	0.85 (0.45 - 1.58)	
Education	High school or less	158	48 (30.4)	1.00	
	Some college	249	75 (30.1)	0.92 (0.63 - 1.33)	
	College graduate	120	39 (32.5)	1.14 (0.73 - 1.77)	
	Graduate studies	139	28 (20.1)	0.65 (0.40 - 1.07)	
Insurance	Yes	618	180 (29.1)	1.00	
	No	25	8 (32.0)	1.04 (0.51 - 2.12)	
	Missing	23	2 (8.7)	-	
**Health-related factors**					
Body mass index prior to diagnosis (kg/m^2^)	<25	306	70 (22.9)	1.00	1.00
	25-29.9	220	67 (30.5)	1.40 (0.99 - 1.96)	1.25 (0.88 - 1.76)
	30+	140	53 (37.9)	1.83 (1.27 - 2.63)	1.59 (1.09 - 2.31)
	Per unit of BMI			1.04 (1.02 - 1.07)	1.04 (1.01 - 1.06)
				*P trend < 0.001*	*P trend = 0.01*
Menopausal status^d^	Premenopausal	259	82 (31.7)	1.00	
	Postmenopausal	341	80 (23.5)	0.81 (0.51 - 1.28)	
	Unknown	66	28 (42.4)	1.44 (0.91 - 2.26)	
Hypertension	No	468	122 (26.1)	1.00	1.00
	Yes	198	68 (34.3)	1.54 (1.11 - 2.12)	1.49 (1.06 - 2.10)
Diabetes	No	607	174 (28.7)	1.00	
	Yes	59	16 (27.1)	0.96 (0.57 - 1.62)	
Arthritis	No	480	135 (28.1)	1.00	
	Yes	186	55 (29.6)	1.19 (0.86 - 1.65)	
Charlson Comorbidity Index	0	599	177 (29.6)	1.00	
	1-2	65	13 (20.0)	0.70 (0.40 - 1.24)	
	Missing	2	0 (0.0)	-	
**Hormonal factors**					
Oral contraceptives use prior to diagnosis	No	189	48 (25.4)	1.00	
	Yes	477	142 (29.8)	1.12 (0.79 - 1.58)	
Estrogen use prior to diagnosis^d,e^	No	120	26 (21.7)	1.00	
	Yes	210	52 (24.8)	1.36 (0.83 - 2.24)	
	Missing	11	2 (18.2)	-	
Progestin use prior to diagnosis^d,e^	No	205	52 (25.4)	1.00	
	Yes	119	23 (19.3)	0.94 (0.56 - 1.59)	
	Missing	17	5 (29.4)	-	
**Lifestyle factors**					
Pack-years of smoking	<100 cigarettes in lifetime, <0.05 pack-years	326	92 (28.2)	1.00	
	0.05-5.3 pack-years	112	33 (29.5)	1.06 (0.71 - 1.58)	
	5.4-20.5 pack-years	111	37 (33.3)	1.31 (0.89 - 1.93)	
	>20.5 pack-years	114	27 (23.7)	0.93 (0.60 - 1.44)	
	Missing	3	1 (33.3)	-	
	Per pack-year of smoking			1.00 (0.99 - 1.01)	
				*P trend = 0.47*	
Alcohol intake year prior to diagnosis (grams per day)	<1	329	102 (31.0)	1.00	
	1-6	99	25 (25.3)	0.82 (0.52 - 1.28)	
	>6	117	29 (24.8)	0.94 (0.61 - 1.45)	
	Missing	121	34 (28.1)	1.28 (0.79 - 2.09)	
	Per gram of alcohol			0.99 (0.97 - 1.01)	
				*P trend = 0.42*	
Sports and recreational activities year prior to diagnosis (MET hours/week)	<0.5	223	68 (30.5)	1.00	
	0.5-20.0	298	85 (28.5)	1.13 (0.80 - 1.59)	
	>20.0	144	37 (25.7)	1.09 (0.71 - 1.67)	
	Missing	1	0 (0.0)	-	
	Per MET hour			1.00 (0.99 - 1.01)	
				*P trend = 0.80*	

^a^HR is adjusted for race/ethnicity (non-Hispanic white (New Mexico), non-Hispanic white (Western Washington), black (Los Angeles), Hispanic (New Mexico)) and age at diagnosis (35-44, 45-49, 50-54, 55-59, 60-64). Age is adjusted for only race/ethnicity. Race/ethnicity is adjusted for only age. ^b^Cox proportional hazard model includes age, race/ethnicity, surgery type, number of excised lymph nodes, chemotherapy, BMI, and hypertension. ^c^‘1 to 9’ category includes ‘at least one lymph node removed’. ^d^Age in years (35 to 64) was used to adjust for the age effect. ^e^Only postmenopausal women were included. N, number; LE, lymphedema; HR, hazard ratio; CI, confidence interval; NOS, not otherwise specified; NM, New Mexico; WW, Western Washington; LA, Los Angeles County; BMI, body mass index; MET, metabolic equivalent task.

### Multivariable analyses overall

Tumor size and tamoxifen did not significantly improve the model fit once number of excised lymph nodes was in the model, and thus these variables were not included in the final multivariable model. The final model included age at diagnosis, race/ethnicity, surgery type, number of excised lymph nodes, chemotherapy, BMI, and hypertension. In this multivariable model, the 95% CI for black women no longer excluded one (versus non-Hispanic white women; HR = 1.16, 95% CI: 0.83 to 1.61). Treatment-related factors including total or modified radical mastectomy (versus partial or less than total mastectomy; HR = 1.37, 95% CI: 1.01 to 1.85), no lymph nodes removed (versus 10 or more excised lymph nodes; HR = 0.17, 95% CI: 0.08 to 0.33), and chemotherapy (HR = 1.48, 95% CI: 1.09 to 2.02) were associated with the risk of lymphedema. The risk of developing lymphedema increased by 5% for each lymph node removed (*P* trend <0.001). Among the health-related factors, having BMI ≥30 kg/m^2^ (versus BMI <25 kg/m^2^; HR = 1.59, 95% CI: 1.09 to 2.31) and having hypertension (HR = 1.49, 95% CI: 1.06 to 2.10) were associated with increased lymphedema risk. We observed a statistically significant increasing trend in risk with increasing BMI (*P* trend = 0.01). None of the prediagnosis lifestyle factors had a substantial effect on the risk of lymphedema. The results from the multivariable analyses were not meaningfully changed when we performed sensitivity analyses in which we excluded women with *in situ* breast cancer or when we reran analyses censoring women who developed a recurrence or new primary breast cancer at the time of these events.

### Multivariable analyses by timing of lymphedema onset

Women’s characteristics and their associations with early-onset and late-onset lymphedema are presented in Table 2. Older age was associated with decreased risk of late-onset lymphedema (*P* trend = 0.002), but not with early-onset lymphedema (*P* trend = 0.91). The risk of late-onset lymphedema decreased by 5% per year increase in age (HR = 0.95, 95% CI: 0.92 to 0.98). We observed a statistically significant difference in the age effect between early- and late-onset lymphedema when age was treated as continuous (*P* = 0.02). Our results also showed that the risk of both early-onset and late-onset lymphedema increased with increasing number of excised lymph nodes (HR = 1.04, 95% CI: 1.01 to 1.06; HR = 1.06, 95% CI: 1.03 to 1.09; respectively). The influence of chemotherapy on risk for late-onset lymphedema (HR = 1.77, 95% CI: 1.13 to 2.78) was greater than the effect of chemotherapy on risk for early-onset lymphedema (HR = 1.28, 95% CI: 0.86 to 1.91); however, these two hazard ratios did not differ statistically (*P* = 0.27).

**Table 2:** **Participant characteristics and their associations with early-onset and late-onset lymphedema**

Characteristics	Categories	Early LE/ at risk	Late LE/ at risk	Early-onset LE HR (95% CI)^a^	Late-onset LE HR (95% CI)^a^	***P***for interaction^b^
**Breast cancer and treatment**						
Age at diagnosis (years)	35-44	18/123	25/105	1.00	1.00	
	45-49	22/128	21/106	1.19 (0.63 - 2.24)	0.83 (0.46 - 1.51)	
	50-54	29/175	17/146	1.19 (0.65 - 2.17)	0.51 (0.27 - 0.96)	
	55-59	20/128	15/108	1.04 (0.54 - 2.00)	0.59 (0.31 - 1.12)	
	60-64	16/112	7/96	1.03 (0.50 - 2.10)	0.31 (0.13 - 0.74)	*0.15*
	Per unit of age			1.00 (0.97 - 1.03)	0.95 (0.92 - 0.98)	0.02
				*P trend = 0.91*	*P trend = 0.002*	
Surgery type	Partial/less than total mastectomy/surgery, NOS	57/442	51/385	1.00	1.00	
	Total mastectomy/modified radical mastectomy	48/224	34/176	1.41 (0.95 - 2.09)	1.32 (0.84 - 2.06)	*0.82*
Number of excised lymph nodes	0	5/143	4/138	0.20 (0.08 - 0.51)	0.13 (0.05 - 0.37)	
	1-9^c^	19/162	13/143	0.57 (0.34 - 0.95)	0.36 (0.20 - 0.65)	
	10+	81/361	68/280	1.00	1.00	*0.44*
	Per lymph node			1.04 (1.01 - 1.06)	1.06 (1.03 - 1.09)	*0.20*
				*P trend = 0.003*	*P trend <0.001*	
Chemotherapy	No	47/413	36/366	1.00	1.00	
	Yes	58/253	49/195	1.28 (0.86 - 1.91)	1.77 (1.13 - 2.78)	*0.27*
**Sociodemographic factors**						
Race/ethnicity	Non-Hispanic white (NM, WW)	50/371	38/321	1.00	1.00	
	Black (LA)	45/226	37/181	1.08 (0.70 - 1.65)	1.26 (0.79 - 2.02)	
	Hispanic white (NM)	10/69	10/59	0.90 (0.45 - 1.78)	1.24 (0.61 - 2.50)	*0.77*
**Health-related factors**						
Body mass index prior to diagnosis (kg/m^2^)	<25	37/306	33/269	1.00	1.00	
	25-29.9	36/220	31/184	1.24 (0.78 - 1.98)	1.25 (0.76 - 2.06)	
	30+	32/140	21/108	1.64 (1.00 - 2.67)	1.52 (0.87 - 2.66)	*0.97*
	Per unit of BMI			1.03 (1.00 - 1.07)	1.04 (1.00 - 1.08)	*0.91*
				*P trend = 0.05*	*P trend = 0.07*	
Hypertension	No	63/468	59/405	1.00	1.00	
	Yes	42/198	26/156	1.65 (1.08 - 2.54)	1.30 (0.80 - 2.13)	*0.44*

^a^HR is adjusted for all the other variables in the table. ^b^*P* value for interaction between a characteristic and timing of lymphedema onset (early versus late) is derived from a likelihood ratio test. ^c^‘1 to 9’ category includes ‘at least one lymph node removed’. LE, lymphedema; HR, hazard ratio; CI, confidence interval; NOS, not otherwise specified; NM, New Mexico; WW, Western Washington; LA, Los Angeles County; BMI, body mass index.

### Multivariable analyses stratified by race/ethnicity

The results showed that the associations of chemotherapy and hypertension with the risk of developing lymphedema varied across racial/ethnic groups (*P* = 0.01, *P* <0.001, respectively) (Table 3); both receipt of chemotherapy and hypertension were associated with an elevated risk of lymphedema among black women only (HR = 2.69, 95% CI: 1.61 to 4.50; HR = 2.73, 95% CI: 1.65 to 4.53; respectively). The associations of age at breast cancer diagnosis and surgery type with the risk of lymphedema also appeared to vary across the racial/ethnic groups, but no statistically significant interactions by race/ethnicity were detected (Table 3).

**Table 3:** **Risk factors for arm lymphedema stratified by race/ethnicity**

			Non-Hispanic white		Black				Hispanic white		
Characteristics	Categories	N	LE (%)	HR (95% CI)^a^	N	LE (%)	HR (95% CI)^a^	N	LE (%)	HR (95% CI)^a^	***P***for interaction^b^
Age at diagnosis (years)	35-44	56	19 (33.9)	1.00	53	21 (39.6)	1.00	14	3 (21.4)	1.00	
	45-49	65	16 (24.6)	0.90 (0.45 - 1.77)	46	20 (43.5)	1.00 (0.53 - 1.92)	17	7 (41.2)	2.23 (0.50 - 10.07)	
	50-54	112	28 (25.0)	0.77 (0.42 - 1.40)	48	17 (35.4)	0.87 (0.44 - 1.72)	15	1 (6.7)	0.40 (0.04 - 4.30)	
	55-59	77	17 (22.1)	0.74 (0.38 - 1.44)	37	13 (35.1)	0.69 (0.33 - 1.44)	14	5 (35.7)	2.77 (0.56 - 13.77)	
	60-64	61	8 (13.1)	0.42 (0.17 - 1.00)	42	11 (26.2)	0.60 (0.26 - 1.34)	9	4 (44.4)	9.17 (1.43 - 58.94)	*0.24*
Surgery type	Partial/less than total mastectomy/ surgery, NOS	272	53 (19.5)	1.00	123	43 (35.0)	1.00	47	12 (25.5)	1.00	
	Total/modified radical mastectomy	99	35 (35.4)	1.58 (1.02 - 2.45)	103	39 (37.9)	1.15 (0.72 - 1.81)	22	8 (36.4)	2.16 (0.71 - 6.54)	*0.39*
Number of excised lymph nodes	0	88	3 (3.4)	0.10 (0.03 - 0.32)	41	5 (12.2)	0.41 (0.16 - 1.10)	14	1 (7.1)	0.09 (0.01 - 0.80)	
	1-9^c^	100	18 (18.0)	0.48 (0.28 - 0.82)	49	13 (26.5)	0.64 (0.35 - 1.18)	13	1 (7.7)	0.09 (0.01 - 0.70)	
	10+	183	67 (36.6)	1.00	136	64 (47.1)	1.00	42	18 (42.9)	1.00	*0.48*
Chemotherapy	No	250	43 (17.2)	1.00	124	27 (21.8)	1.00	39	13 (33.3)	1.00	
	Yes	121	45 (37.2)	1.25 (0.80 - 1.95)	102	55 (53.9)	2.69 (1.61 - 4.50)	30	7 (23.3)	0.69 (0.24 - 2.03)	0.01
Body mass index (kg/m^2^)	<25	192	42 (21.9)	1.00	89	23 (25.8)	1.00	25	5 (20.0)	1.00	
	25-29.9	114	26 (22.8)	1.05 (0.64 - 1.73)	76	32 (42.1)	1.58 (0.90 - 2.78)	30	9 (30.0)	2.25 (0.65 - 7.72)	
	30+	65	20 (30.8)	1.50 (0.86 - 2.61)	61	27 (44.3)	1.65 (0.91 - 2.98)	14	6 (42.9)	3.94 (0.99 - 15.75)	*0.46*
Hypertension	No	292	73 (25.0)	1.00	119	30 (25.2)	1.00	57	19 (33.3)	1.00	
	Yes	79	15 (19.0)	0.95 (0.52 - 1.74)	107	52 (48.6)	2.73 (1.65 - 4.53)	12	1 (8.3)	0.21 (0.02 - 2.00)	<0.001

^a^Race/ethnicity-specific HR is adjusted for all of the other variables in this table. ^b^*P* value for interaction between a characteristic and race/ethnicity is derived from a likelihood ratio test. ^c^‘1 to 9’ category includes ‘at least one lymph node removed’. LE, lymphedema; HR, hazard ratio; CI, confidence interval; NOS, not otherwise specified.

## Discussion

This prospective cohort study of women diagnosed with first primary *in situ* or Stage I to IIIA invasive breast cancer between the ages of 35 and 64 years supports previous findings that the risk of lymphedema is higher among women who had more lymph nodes removed, more extensive surgery, and higher BMI. This study also highlights the importance of long-term monitoring of breast cancer survivors, particularly those who are younger, have had more lymph nodes removed, or received chemotherapy, as they are at a higher risk of developing late-onset lymphedema.

This study demonstrates that the cumulative incidence of lymphedema increases with time and that lymphedema can develop later in the survival trajectory. Although many studies have reported incidence of lymphedema, comparison across studies is difficult because of variability in lymphedema definition and assessment, length of follow-up, and associated patient characteristics. In a study conducted by Kwan and colleagues [17] where length of follow-up for each participant was considered to calculate cumulative incidence of lymphedema, the cumulative incidence of lymphedema was 10.4% at one year and 13.5% at two years after breast cancer diagnosis. These cumulative incidence values were lower than those observed in our study, perhaps because their participants were diagnosed with breast cancer more recently when sentinel lymph node biopsy was more common than it was when participants in the HEAL Study were diagnosed. Also, the study by Kwan *et al*. required hospital records to establish the presence of lymphedema.

The existing data on whether lymphedema incidence varies by age have been inconsistent. Contrary to some of the previous studies [16],[18] and despite the limited age range in our study (35 to 64 years), we found that older women were less likely to develop lymphedema than younger women. We observed a statistically significant, decreasing linear trend in lymphedema risk associated with increasing age after adjusting for breast cancer characteristics and treatment factors, hypertension, and BMI (*P* = 0.04). Our results for age are consistent with those from other studies [19],[29],[40]. When we examined the effects of age by timing of onset, we found that age was inversely associated with risk of late-onset lymphedema, but not with risk of early-onset lymphedema. The age-by-time interaction was statistically significant when age was treated as continuous (*P* = 0.02). The reason why older women are less likely to develop lymphedema later in the survival trajectory is unclear. A previous study showed that infection or injury was associated with increased risk of late-onset lymphedema [20]. We were unable to adjust for the effect of infection or injury. Thus we cannot rule out the possibility of residual confounding. More research is needed to study the age effect on late-onset lymphedema.

This study identified two modifiable risk factors for lymphedema. One of the modifiable risk factors was prediagnosis BMI; women with higher prediagnosis BMI were at a higher risk of lymphedema. The association between prediagnosis BMI and risk of lymphedema has been shown in the Iowa Women’s Health Study [22] and another study conducted by Jammallo *et al.*[32]. Other studies have found an association between at-diagnosis or post-diagnosis BMI and the risk of lymphedema [15],[23],[24],[28]. The mechanism by which excess weight increases the risk of lymphedema remains unclear; the risk of lymphedema may be elevated in obese women due to additional demand on both the vascular and lymphatic systems to transport fluid [28]. Weight gain during survivorship in relation to lymphedema risk is also of interest. Petrek *et al*. [20] reported an association between weight gain since operation and risk of breast cancer-related lymphedema. However, our study failed to demonstrate an association between change in BMI (BMI at 30-month assessment minus prediagnosis BMI) and late-onset lymphedema (data not shown). More studies are needed to understand the association between BMI and the risk of lymphedema.

The other modifiable risk factor we found in the present study was hypertension. We found that hypertension was associated with increased risk of lymphedema after adjusting for BMI, which contradicts some studies where no association between hypertension and lymphedema was found [21]-[23],[27]. A possible mechanism by which hypertension increases the risk is through increased capillary filtration due to elevated hydrostatic pressure. When capillary filtration increases and lymphatic drainage is insufficient, the fluid accumulates in interstitial space leading to swelling of the arm. Our data indicated that the risk of lymphedema among women with hypertension, relative to women without hypertension, was not altered substantially after adjusting for antihypertensive medication use (HR = 1.58, 95% CI: 1.05 to 2.37). We also examined potential confounding effects of different types of antihypertensive medications such as vasodilators, calcium blockers, beta blockers, alpha blockers, angiotensin-converting enzyme, and diuretics. Among these medications, only the use of diuretics substantially decreased the HR associated with hypertension; the 95% CI included one after adjusting for diuretics use (HR = 1.40, 95% CI: 0.96 to 2.03). Our study also showed that hypertension increased the risk of lymphedema among black women, but not other women. Since black women with hypertension were more likely to take diuretics than non-black women with hypertension in our study (*P* = 0.02), the observed interaction by race/ethnicity could possibly be explained by the association between use of diuretics and the risk of lymphedema. However, among women with hypertension, we found no significant association between diuretic use and lymphedema (treatment with diuretics versus no treatment with diuretics; HR = 1.04, 95% CI: 0.61 to 1.78). Similarly, we found no significant association between diuretic use and lymphedema among black women with hypertension (treatment with diuretics versus no treatment with diuretics; HR = 1.06, 95% CI: 0.56 to 2.00). These findings do not support the hypothesis that the interaction between hypertension and race is due to the more frequent use of diuretics among black women. Due to lack of information on duration, timing, and rationale for selecting a particular type of antihypertensive medication in this study, the association between antihypertensive medications and lymphedema risk needs further clarification in future studies.

Our study results agree with the majority of studies showing that lymphedema risk increases with increasing number of excised lymph nodes [12],[17],[22],[25]. This positive association between number of excised lymph nodes and risk remained significant after restricting the cohort to women with invasive breast cancer (*P* trend = 0.002). On the other hand, our study failed to confirm an association with radiation therapy. We also did not observe effect modification of radiation therapy by number of excised lymph nodes (*P* = 0.18). However, detailed information on location, dose, and duration of radiation therapy was not available in this study. Since the risk of lymphedema depends on the type of radiotherapy [41], we may have missed an association with radiation treatment.

Evidence on associations between chemotherapy and lymphedema risk has been inconsistent, with some studies finding a positive association [8],[12],[17],[19],[22],[23] and others finding no association [16],[25]-[27],[29]. The present study showed a positive association between chemotherapy and overall lymphedema risk after adjusting for other risk factors (HR = 1.48, 95% CI: 1.09 to 2.02). This association persisted after excluding women with *in situ* breast cancer (HR = 1.47, 95% CI: 1.06 to 2.03). When we examined individual types of chemotherapy adjusting for other risk factors, we found that 5-fluorouracil, methotrexate, and cyclophosphamide were associated with increased risk of lymphedema whereas taxane and doxorubicin were not. Women who received 5-fluorouracil, methotrexate, and cyclophosphamide in combination may have a higher risk of infection because these agents together tend to reduce the number of white blood cells and compromise immune response [42]. It is conceivable that the elevated lymphedema risk observed in women who received these chemotherapy agents may be partly attributable to infection. Our exploratory analysis further demonstrated a statistically significant interaction between chemotherapy and hypertension in relation to lymphedema risk among women diagnosed with invasive breast cancer. More specifically, the risk of lymphedema was elevated only among women with hypertension who received chemotherapy (versus women without hypertension who did not receive chemotherapy; HR = 2.36, 95% CI: 1.54 to 3.64). Swystun and colleagues [43] suggested that the chemotherapy metabolite acrolein may upregulate procoagulant pathways, while impairing endogenous anticoagulant pathways. In our study, 222 (88%) of 253 women treated with chemotherapy received cyclophosphamide, which has been associated with an increased risk of thrombosis [43]-[45]. It is possible that this procoagulant activity might overburden the lymphatic system in women with preexisting hypertension.

Although lymphedema among breast cancer survivors has been studied extensively, data from studies with long-term follow-up of ethnically diverse populations remain scarce. Kwan *et al.*[17] found that black women had a higher risk of lymphedema compared to white women whereas Meeske *et al*. [2] found no association between race (black versus non-Hispanic white) and lymphedema risk after adjusting for other risk factors. Note that the black women in the study by Meeske *et al*. [2] are the same black women who are included in this report, but follow-up for lymphedema was limited to the 40-month assessment in their study. With follow-up extended through the 123-month assessment, we observed that the elevated lymphedema risk among black women was attenuated after adjusting for age, treatment-related factors, BMI, and hypertension (versus non-Hispanic white women; HR = 1.16, 95% CI: 0.83 to 1.61). Our exploratory analysis examining racial/ethnic-specific associations showed that chemotherapy and hypertension were lymphedema risk factors only among black breast cancer survivors; thus, the observed overall effects of these factors on lymphedema risk appear to be limited to black women.

In our study, the median length of follow-up since breast cancer diagnosis was 10.2 years; this is longer than the median length of follow-up in many prior breast cancer-related lymphedema studies. With this extended follow-up time, we were able to capture a substantial number of cases of late-onset lymphedema, which allowed us to compare risk factors between early- and late-onset lymphedema. Moreover, unlike many previous studies, this study consisted of a large, multiethnic sample of breast cancer survivors drawn from population-based cancer registries. This diverse population allowed us to study associations within groups defined by race and ethnicity and identify potential heterogeneity across race and ethnicity. However, this study has several limitations, which should be considered when interpreting the results. This study consisted of women who were diagnosed between 1995 and 1999 when sentinel lymph node biopsy was less common. According to Chen *et al*. [46], the use of sentinel lymph node biopsy increased from 26.8% in 1998 to 65.5% in 2005 among early-stage breast cancer patients. Given the risk of lymphedema is smaller when sentinel lymph node biopsy is used instead of axillary lymph node dissection, our estimate of lymphedema incidence may be greater than the incidence observed in more recent years. Furthermore, the selection of chemotherapeutic agents has expanded over the years, possibly limiting the application of results to more recent regimens. In our study, lymphedema was reported at two time points and women indicated the approximate date when they first experienced lymphedema, but we did not routinely monitor the status of lymphedema over the follow-up period. Furthermore, this study relied solely on self-report to define existence of lymphedema and the self-report was not verified using medical records. However, both sensitivity and specificity of self-reported lymphedema were found to be high in a previous study [47], and our results are largely consistent with those from studies where objective measurements of lymphedema were used. Our results were consistent with those showing associations of axillary lymph node excision [16],[25],[31], greater BMI [27],[30],[31], and extensive surgery [16],[30],[31] with increased risk of lymphedema, while the prior associations of age [16],[25]-[27],[30],[31], radiotherapy [25]-[27], and chemotherapy [16],[25]-[27] with lymphedema were somewhat inconsistent with our data. Inconsistent findings across studies could be explained by differences in demographic and tumor characteristics, or variations in length of follow-up. We were also limited by insufficient statistical power to study three-way interactions; for example, we were unable to determine how race/ethnicity, chemotherapy, and hypertension interact in relation to lymphedema risk.

## Conclusions

Our multiethnic cohort study confirms that lymphedema incidence is highest in the first year following breast cancer diagnosis, but also indicates that approximately 45% of women who developed lymphedema first experienced the condition more than one year after initial diagnosis. Furthermore, it shows that the majority of lymphedema cases persist for a long time. It is important to pay closer attention to women who had extensive lymph node dissection, had more extensive surgery, or were obese prior to diagnosis in order to detect lymphedema and provide education and treatment to manage lymphedema at an early stage. More data from multiethnic cohort studies are needed to confirm our finding that hypertension and chemotherapy are risk factors only among black women. Clinical trials are needed to determine whether treatment for hypertension and obesity might prevent the incidence or severity of lymphedema in breast cancer survivors.

## Additional file

## Electronic supplementary material

Additional file 1: Table S1: Participant characteristics by race/ethnicity. (DOC 110 KB)

Below are the links to the authors’ original submitted files for images.

Authors’ original file for figure 1

Authors’ original file for figure 2

## Abbreviations

BMI: body mass index
CI: confidence interval
HEAL: Health, Eating, Activity, and Lifestyle
HR: hazard ratio
LA: Los Angeles county
LE: lymphedema
MET: metabolic equivalent task
NM: New Mexico
NOS: not otherwise specified
SEER: surveillance, epidemiology, and end results
WW: Western Washington
