# Supplementary material for: Risk factors for self-reported arm lymphedema among female breast cancer survivors: a prospective cohort study
Source: Breast Cancer Res. 2014 Aug 22;16:414. doi: 10.1186/s13058-014-0414-x (PMC4189147; doi:10.1186/s13058-014-0414-x)
Supplement: Supplementary file 1 — Additional file 1: Table S1: Participant characteristics by race/ethnicity. (DOC 110 KB) [file 13058_2014_414_MOESM1_ESM.doc]

**Table S1 Participant characteristics by race/ethnicity**

| **Characteristics** | **Categories** | **Non-Hispanic white**  **(N=371)**  **N (%)** | **Black**  **(N=226)**  **N (%)** | **Hispanic white**  **(N=69)**  **N (%)** |
| --- | --- | --- | --- | --- |
| **Breast cancer and treatment** |  |  |  |  |
| Age at diagnosis (years) | 35–44 | 56 (15.1) | 53 (23.5) | 14 (20.3) |
|  | 45–49 | 65 (17.5) | 46 (20.4) | 17 (24.6) |
|  | 50–54 | 112 (30.2) | 48 (21.2) | 15 (21.7) |
|  | 55–59 | 77 (20.8) | 37 (16.4) | 14 (20.3) |
|  | 60–64 | 61 (16.4) | 42 (18.6) | 9 (13.0) |
|  | Mean (standard deviation) | 52.2 (6.7) | 50.8 (7.8) | 50.8 (7.4) |
| Disease stage | *In situ* | 96 (25.9) | 47 (20.8) | 12 (17.4) |
|  | Localized | 206 (55.5) | 98 (43.4) | 42 (60.9) |
|  | Regional | 69 (18.6) | 81 (35.8) | 15 (21.7) |
| Tumor size (mm) | <10 | 113 (30.5) | 45 (19.9) | 20 (29.0) |
|  | 10-19 | 125 (33.7) | 66 (29.2) | 25 (36.2) |
|  | 20+ | 101 (27.2) | 86 (38.1) | 21 (30.4) |
|  | Missing | 32 (8.6) | 29 (12.8) | 3 (4.4) |
| Cancer location | Nipple, central portion, upper/lower inner quadrant | 84 (22.6) | 43 (19.0) | 16 (23.2) |
|  | Upper/lower outer quadrant | 163 (43.9) | 99 (43.8) | 30 (43.5) |
|  | Overlapping lesion | 84 (22.6) | 47 (20.8) | 16 (23.2) |
|  | Axillary tail/NOS | 40 (10.8) | 37 (16.4) | 7 (10.1) |
| Surgery type | Partial/less than total mastectomy/surgery, NOS | 272 (73.3) | 123 (54.4) | 47 (68.1) |
|  | Total mastectomy/modified radical mastectomy | 99 (26.7) | 103 (45.6) | 22 (31.9) |
| Reconstructive surgery | No | 304 (81.9) | 180 (79.7) | 49 (71.0) |
|  | Yes | 56 (15.1) | 36 (15.9) | 13 (18.8) |
|  | Missing | 11 (3.0) | 10 (4.4) | 7 (10.1) |
| Number of excised lymph nodes | 0 | 88 (23.7) | 41 (18.1) | 14 (20.3) |
|  | 1–9^a^ | 100 (27.0) | 49 (21.7) | 13 (18.8) |
|  | 10+ | 183 (49.3) | 136 (60.2) | 42 (60.9) |
| Radiation | No | 126 (34.0) | 122 (54.0) | 22 (31.9) |
|  | Yes | 245 (66.0) | 104 (46.0) | 47 (68.1) |
| Chemotherapy | No | 250 (67.4) | 124 (54.9) | 39 (56.5) |
|  | Yes | 121 (32.6) | 102 (45.1) | 30 (43.5) |
| Tamoxifen | No | 196 (52.8) | 149 (65.9) | 37 (53.6) |
|  | Yes | 175 (47.2) | 77 (34.1) | 32 (46.4) |

**Table S1 Participant characteristics by race/ethnicity *(continued)***

| **Characteristics** | **Categories** | **Non-Hispanic white**  **(N=371)**  **N (%)** | **Black**  **(N=226)**  **N (%)** | **Hispanic white**  **(N=69)**  **N (%)** |
| --- | --- | --- | --- | --- |
| **Sociodemographic factors** |  |  |  |  |
| Marital status | Married | 262 (70.6) | 137 (60.6) | 46 (66.7) |
|  | Widowed | 12 (3.2) | 17 (7.5) | 5 (7.3) |
|  | Divorced/separated | 67 (18.1) | 57 (25.2) | 16 (23.2) |
|  | Never married | 30 (8.1) | 15 (6.6) | 2 (2.9) |
| Education | High school or less | 54 (14.6) | 77 (34.1) | 27 (39.1) |
|  | Some college | 125 (33.7) | 101 (44.7) | 23 (33.3) |
|  | College graduate | 86 (23.2) | 27 (12.0) | 7 (10.1) |
|  | Graduate studies | 106 (28.6) | 21 (9.3) | 12 (17.4) |
| Insurance | Yes | 345 (93.0) | 207 (91.6) | 66 (95.7) |
|  | No | 8 (2.2) | 14 (6.2) | 3 (4.3) |
|  | Missing | 18 (4.9) | 5 (2.2) | 0 (0.0) |
| **Health-related factors** |  |  |  |  |
| Body mass index prior to diagnosis (kg/m^2^) | <25 | 192 (51.8) | 89 (39.4) | 25 (36.2) |
|  | 25-29.9 | 114 (30.7) | 76 (33.6) | 30 (43.5) |
|  | 30+ | 65 (17.5) | 61 (27.0) | 14 (20.3) |
| Menopausal status | Premenopausal | 143 (38.5) | 89 (39.4) | 27 (39.1) |
|  | Postmenopausal | 196 (52.8) | 111 (49.1) | 34 (49.3) |
|  | Unknown | 32 (8.6) | 26 (11.5) | 8 (11.6) |
| Hypertension | No | 292 (78.7) | 119 (52.7) | 57 (82.6) |
|  | Yes | 79 (21.3) | 107 (47.3) | 12 (17.4) |
| Diabetes | No | 348 (93.8) | 195 (86.3) | 64 (92.8) |
|  | Yes | 23 (6.2) | 31 (13.7) | 5 (7.2) |
| Arthritis | No | 271 (73.0) | 164 (72.6) | 45 (65.2) |
|  | Yes | 100 (27.0) | 62 (27.4) | 24 (34.8) |
| Charlson Comorbidity Index | 0 | 337 (90.8) | 199 (88.1) | 63 (91.3) |
|  | 1–2 | 32 (8.6) | 27 (11.9) | 6 (8.7) |
|  | Missing | 2 (0.5) | 0 (0.0) | 0 (0.0) |
| **Hormonal factors** |  |  |  |  |
| Oral contraceptives use prior to diagnosis | No | 86 (23.2) | 81 (35.8) | 22 (31.9) |
|  | Yes | 285 (76.8) | 145 (64.2) | 47 (68.1) |
| Estrogen use prior to diagnosis^b^ | No | 44 (22.4) | 64 (57.7) | 12 (35.3) |
|  | Yes | 142 (72.4) | 46 (41.4) | 22 (64.7) |
|  | Missing | 10 (5.1) | 1 (0.9) | 0 (0.0) |
| Progestin use prior to diagnosis^b^ | No | 89 (45.4) | 92 (82.9) | 24 (70.6) |
|  | Yes | 92 (46.9) | 18 (16.2) | 9 (26.5) |
|  | Missing | 15 (7.7) | 1 (0.9) | 1 (2.9) |
| **Lifestyle factors** |  |  |  |  |
| Pack-years of smoking | <100 cigarettes in lifetime, <0.05 pack-years | 183 (49.3) | 110 (48.7) | 33 (47.8) |
|  | 0.05-5.3 | 58 (15.6) | 38 (16.8) | 16 (23.2) |
|  | 5.4–20.5 | 55 (14.8) | 45 (19.9) | 11 (15.9) |
|  | >20.5 | 72 (19.4) | 33 (14.6) | 9 (13.0) |
|  | Missing | 3 (0.8) | 0 (0.0) | 0 (0.0) |
| Alcohol intake year prior to diagnosis (grams per day) | <1 | 137 (36.9) | 170 (75.2) | 22 (31.9) |
|  | 1–6 | 58 (15.6) | 29 (12.8) | 12 (17.4) |
|  | >6 | 86 (23.2) | 25 (11.1) | 6 (8.7) |
|  | Missing | 90 (24.3) | 2 (0.9) | 29 (42.0) |
| Sports and recreational activities year prior to diagnosis  (MET hours/week) | <0.5 | 75 (20.2) | 129 (57.1) | 19 (27.5) |
|  | 0.5–20.0 | 192 (51.8) | 71 (31.4) | 35 (50.7) |
|  | >20.0 | 103 (27.8) | 26 (11.5) | 15 (21.7) |
|  | Missing | 1 (0.3) | 0 (0.0) | 0 (0.0) |

Abbreviations: N, number; NOS, not otherwise specified; MET, metabolic equivalent task

^a^ “1 to 9” category includes “at least one lymph node removed.”

^b^ Only postmenopausal women were included.
