# Supplementary figures and images for: Risk factors for self-reported arm lymphedema among female breast cancer survivors: a prospective cohort study
Source: Breast Cancer Res. 2014 Aug 22;16:414. doi: 10.1186/s13058-014-0414-x (PMC4189147; doi:10.1186/s13058-014-0414-x)

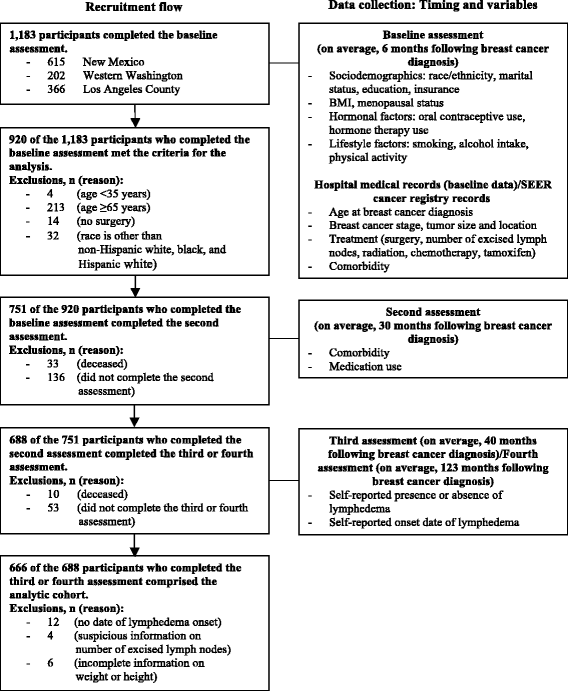

Supplement: Supplementary file 2 — Authors’ original file for figure 1 [file 13058_2014_414_MOESM2_ESM.gif]

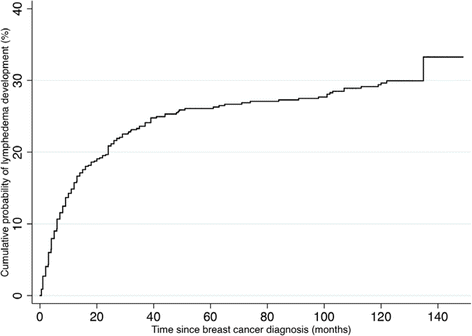

Supplement: Supplementary file 3 — Authors’ original file for figure 2 [file 13058_2014_414_MOESM3_ESM.gif]
